# How flies turn food into progeny

**DOI:** 10.7554/eLife.51289

**Published:** 2019-10-01

**Authors:** Thomas Flatt

**Affiliations:** Department of BiologyUniversity of FribourgFribourgSwitzerland

**Keywords:** sex differences, nutrition, reproduction, gene expression, RNASeq, *D. melanogaster*

## Abstract

Sex-optimal diets have different effects on gene expression in female and male flies.

**Related research article** Camus MF, Piper MDW, Reuter M. 2019. Sex-specific transcriptomic responses to changes in the nutritional environment. *eLife* **8**:e47262. doi: 10.7554/eLife.47262

Females and males have evolved different strategies to achieve the same goal: making babies. For example, males usually produce a large number of cheap sperm cells and often display traits that help to maximize the number of successful matings. Females, on the other hand, tend to 'go for quality' by producing a small number of relatively large eggs (each of which requires a lot of energy to produce), and exhibit traits that help them maximize their 'return on investment’. It is thus not surprising that females and males require different diets to maximize their progeny. In various insects, for instance, females rely mainly on protein intake to fuel egg production, whereas males use a higher proportion of carbohydrates to optimize their Darwinian fitness (Lee et al., 2008; Maklakov et al., 2008; Jensen et al., 2015; Camus et al., 2017).

A growing body of work is dedicated to studying the specific dietary requirements that maximize reproductive success of each sex, but the underlying molecular and physiological mechanisms remain poorly understood, especially in males. Now, in eLife, Florencia Camus and Max Reuter from University College London, and Matthew Piper from Monash University, report the results of experiments on the fruit fly *Drosophila melanogaster* which show that male and female flies modify the expression of certain genes differently in response to changes in their diet (Camus et al., 2019).

Camus et al. fed the flies a diet that was reproductively optimal either for their own sex or for the other sex, and then sequenced the RNA of these flies. Comparing the results for female and male flies revealed that different genes had distinct responses to the two diets (Figure 1). Many metabolic or ‘core’ genes had similar expression patterns in female and male flies. However, several smaller groups of genes had transcriptional responses that were sex-specific. These groups include genes that only respond to changes in diet in one sex, and genes that exhibit opposite (antagonistic) responses to the same dietary change in male and female flies.

**Figure 1.**
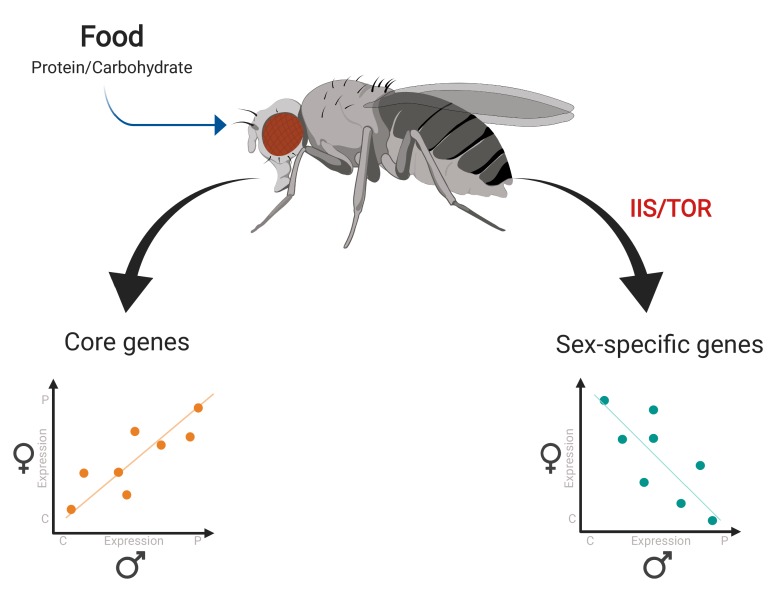
Gene expression responses to changes in diet in female and male fruit flies. Camus et al. examined gene expression in female and male flies given a protein-rich diet (which is optimal for egg production) and a carbohydrate-rich diet (which is optimal for sperm production). Many metabolic genes (‘core genes’) displayed similar responses to diet in both female and male flies (bottom left). A smaller group of genes – including a number of reproductive genes – showed clear-cut differences in expression for each diet depending on sex, with some exhibiting antagonistic behaviors in the two sexes (bottom right). Further analyses revealed that this sex-opposite regulation occurs within the IIS/TOR signaling network.

Among the genes that responded to diet in just one sex, Camus et al. identified genes involved in egg production and hormonal regulation in females, and genes responsible for sperm function in males. One prominent example in this category is *doublesex*, a well-known regulator of sexual differentiation and sex-specific behavior, which showed higher expression in females fed a high-protein diet. Genes with antagonistic responses in males and females include *fit* (*female-specific independent of transformer*), a gene that is upregulated in male flies during courtship and mating. Moreover, the transcripts that showed antagonistic responses between the sexes were enriched for GATA transcription factors which have previously been implicated in nutritional responses (including dietary restriction) and reproductive physiology.

Comparing these results to previously published datasets (Tiebe et al., 2015; Graze et al., 2018) provided compelling bioinformatic evidence that the IIS/TOR signaling network (short for the insulin/insulin-like growth factor signaling/target of rapamycin signaling network) is involved in reproduction. This is particularly interesting given growing evidence that IIS/TOR signaling plays an important role in regulating traits and processes that vary between the sexes. For example, using transcriptional profiling of virgin flies, it has been shown that reducing insulin signaling increases the differences in expression of IIS core pathway genes between the sexes (Graze et al., 2018). The work by Camus et al. makes a significant advance in this area by establishing profound connections between sex-specific dietary optima for reproduction and sex-specific expression changes in IIS/TOR.

To further corroborate the IIS/TOR connection, Camus et al. inhibited TOR signaling with the antagonist rapamycin, leading to disproportionately deleterious effects on reproductive performance when male or female flies were fed their sex-optimal diet. This suggests that TOR signaling is required for the increased reproductive performance conferred by the different diets. These results are consistent with the idea that nutrient-sensing signals mediated by IIS/TOR signaling are somehow inverted between females and males. It will be a fascinating task for future work to uncover how the nutritional signaling inputs into the IIS/TOR network are modulated in a sex-specific way to optimize each sex's reproductive performance.

Together, the results reported by Camus et al. provide fertile ground for future experiments to dissect the mechanisms that underpin sex-specific links between diet, nutrient sensing, metabolism and reproduction.
